# Pigmented Basal Cell Carcinoma Mimicking Melanoma in the Anal Area: A Case Report

**DOI:** 10.1002/ccr3.71982

**Published:** 2026-05-04

**Authors:** Farnaz Araghi, Sareh Salarinejad, Azadeh Rakhshan, Hamideh Moravvej Farshi

**Affiliations:** ^1^ Skin Research Center Shahid Beheshti University of Medical Sciences Tehran Iran; ^2^ Blood Transfusion Research Center High Institute for Research and Education in Transfusion Medicine Tehran Iran; ^3^ Department of Pathology, Shohada‐e‐Tajrish Educational Hospital, School of Medicine Shahid Beheshti University of Medical Sciences Tehran Iran

**Keywords:** basal cell carcinoma, perianal ulcer, pigmented basal carcinoma, skin tumor, ulcer

## Abstract

Early evaluation of persistent perianal lesions is essential even when symptoms appear mild or patients feel embarrassed to seek care. Pigmented basal cell carcinoma in this region is rare and may mimic melanoma clinically. Ongoing histopathological and immunohistochemical assessment is crucial to ensure correct diagnosis and timely surgical management for optimal outcomes.

## 1 Introduction

Basal cell carcinoma (BCC) represents the most common form of skin cancer, typically arising in sun‐exposed areas. Perianal BCC, however, is exceedingly rare, accounting for approximately 0.2% of anorectal malignancies [1].

Basal cell carcinoma occurring in a non‐sun‐exposed region is often associated with Gorlin syndrome. In addition, chronic trauma, prior radiation therapy, or other factors that alter the hedgehog pathway may contribute to the development of sporadic BCC [2].

Only a limited number of BCC subtypes in the anorectal region have been reported without identifiable risk factors (Table 1), with the nodular type being the most common.

**TABLE 1:** Data of included studies.

Author	Year	Gender	Tumor size(cm)	BCC type	Related Risk Factor	Presentation	Location to the anus
Li [1]	2025	M 53	2.5* 3.5	Infiltrative	Sigmoid adenocarcinoma	Ulcer, pruritus	11 o'clock
Fernandez‐Martinez [3]	2018	F 78	3	Nodular	None	Bleeding, pruritus	1 o'clock
Matsumura [4]	2020	F 71	2.5* 3	Nodular	Primary biliary cirrhosis	Growing mass	9 o'clock
Matsumura [4]	2020	F 80	Not reported	Superficial	None	Pruritus	5 o'clock
Takahashi [5]	2020	M 83	1	Nodular	Perianal extramammary paget(co‐occur), Hepatitis C‐type, and hepatocellular carcinoma	Bleeding	3 o'clock
Bulur [6]	2015	M 34	3*6	Nodular	None	Bleeding	7 o'clock
Carr [7]	2018	M 66	3	Pigmented	None	Abscess	9 o'clock
Aldana [8]	2019	M 89	0.5	Nodular	Gastric lymphoma	None	Right side
Espana [9]	1992	F 72	1.5	Nodular	Pelvic radiotherapy	Growing mass	5 o'clock
Karim [10]	2001	M 83	4*3	Nodular	None	Ulcer	7 o'clock
Kort [11]	1995	M 84	5	Nodular	None	Growing mass	7 o'clock
Kreuter [12]	2012	M 88	3*1	Nodular	Prostate cancer	Ulcer	3 o'clock
Riverra‐ Chavarria [13]	2016	F 93	4.5*3.2	Ulcerative	None	Bleeding ulcer	12 o'clock
Manheim [14]	1955	M 72	3*2.5	Pigmented	Fistula tract	Drainage	Anterolateral
Meeks [15]	2019	M 49	2*1	Nodular	None	Bleeding	3 o'clock
Misago [16]	2004	F 88	3.5*2	Nodular	None	Growing mass	9 o'clock
Fuentes‐Calvo [17]	2024	M 59	1.6*2.2	Pigmented	None	Bleeding	9 o'clock
Rosenthal [18]	1967	M 70	2*2	Infiltrative	Epitheliomatosis	Bleeding	6 o'clock
Rosenthal [18]	1967	M 82	3*3	Pigmented	Sigmoid adenocarcinoma	Bleeding	7 o'clock
Shaikh [19]	2010	M 72	5*3.5	Nodular	Past hemorrhoidectomy	Bleeding	10 o'clock
Montagliani [20]	2004	M 66	2	Nodular	None	Bleeding	5 o'clock

Herein, we report a rare case of pigmented BCC presenting as a chronic perianal ulcer.

## 2 Case History

A 57‐year‐old female presented with a chronic, non‐painful ulcer near the anus. Due to embarrassment, she had delayed seeking medical attention. She had no relevant medical or surgical history.

**FIGURE 1:**
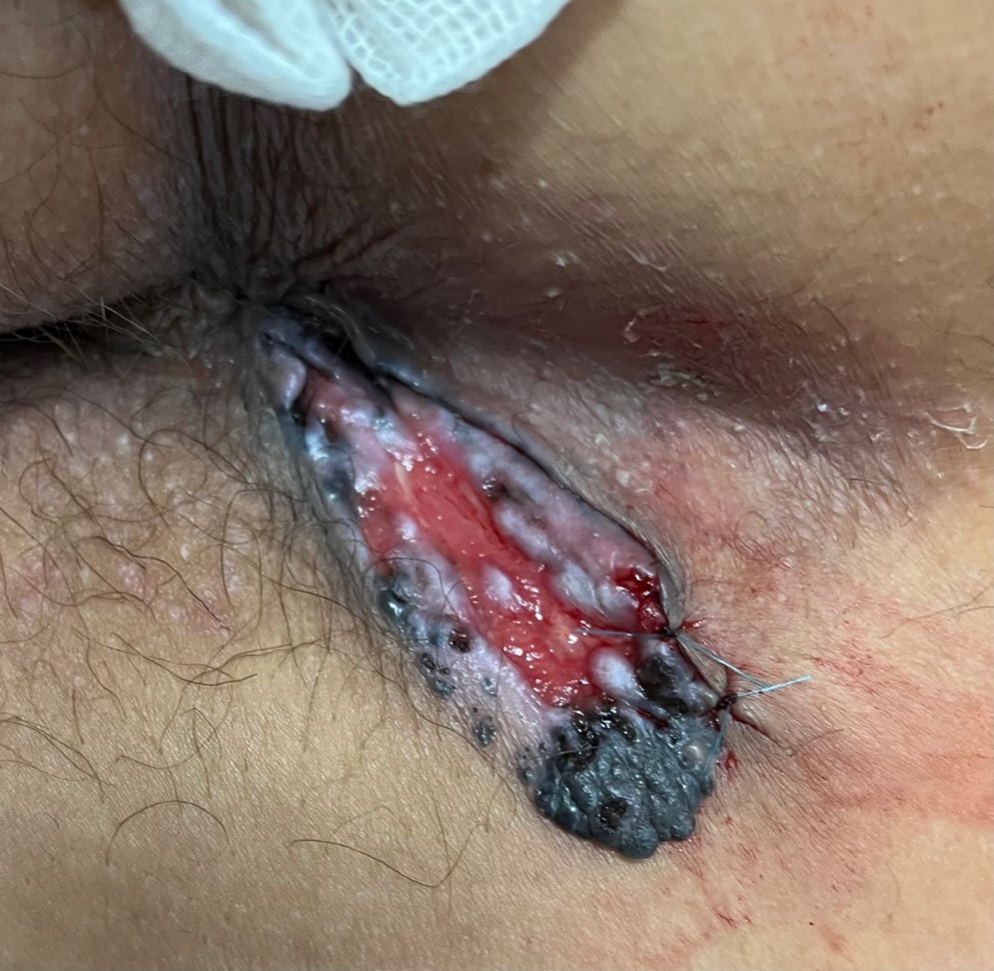
Pigmented Basal Cell Carcinoma mimicking melanoma in the anal area.

On physical examination, an irregular ulcerative lesion with surrounding black pigmentation adjacent to the anus was revealed (Figure 1). The lesion measured approximately 6 cm, was oval with raised margins, and extended from the lateral aspect of the anus. No inguinal lymphadenopathy or rectal masses were detected.

## 3 Differential Diagnosis, Investigations, and Treatment

A wedge skin biopsy was obtained from the pigmented margin of the ulcer, with melanoma considered as the primary differential diagnosis.

**FIGURE 2:**
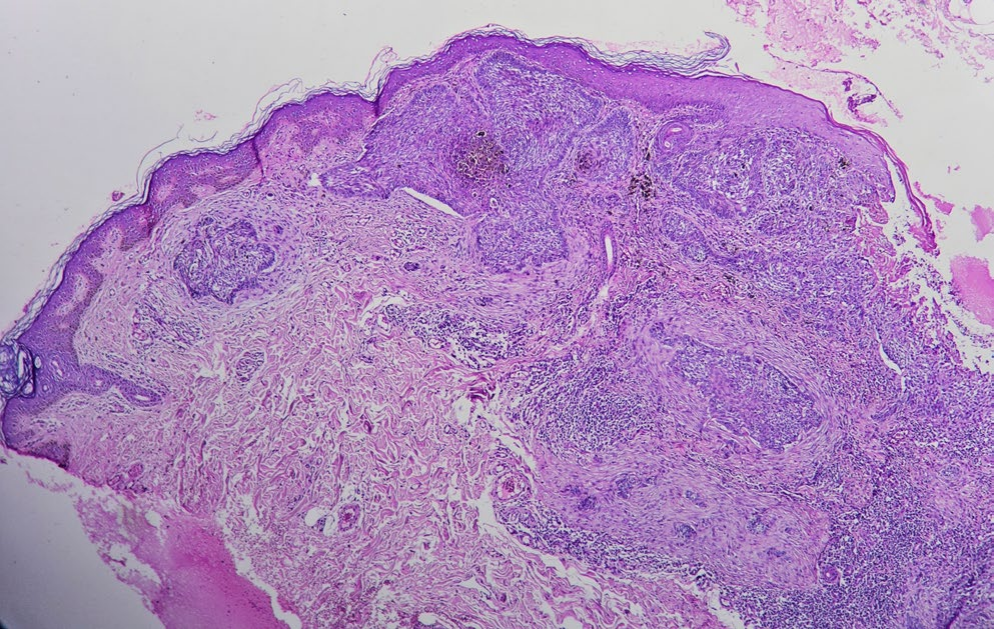
Basaloid cell nests with peripheral palisading (H&Ex40).

The microscopic analysis revealed a dermal basaloid neoplasm with focal epidermal attachment, peripheral palisading, peritumoral mucinous change, cleft formation, focal clear cell changes in nodular pattern, and foci of infiltrative features. Pigment deposition in the tumoral area and adjacent dermis is seen. The overlying epidermis shows focal ulceration, focal parakeratosis, and acanthosis of the adjacent epidermis, without evidence of melanocytic proliferation (Figure 2).

**FIGURE 3:**
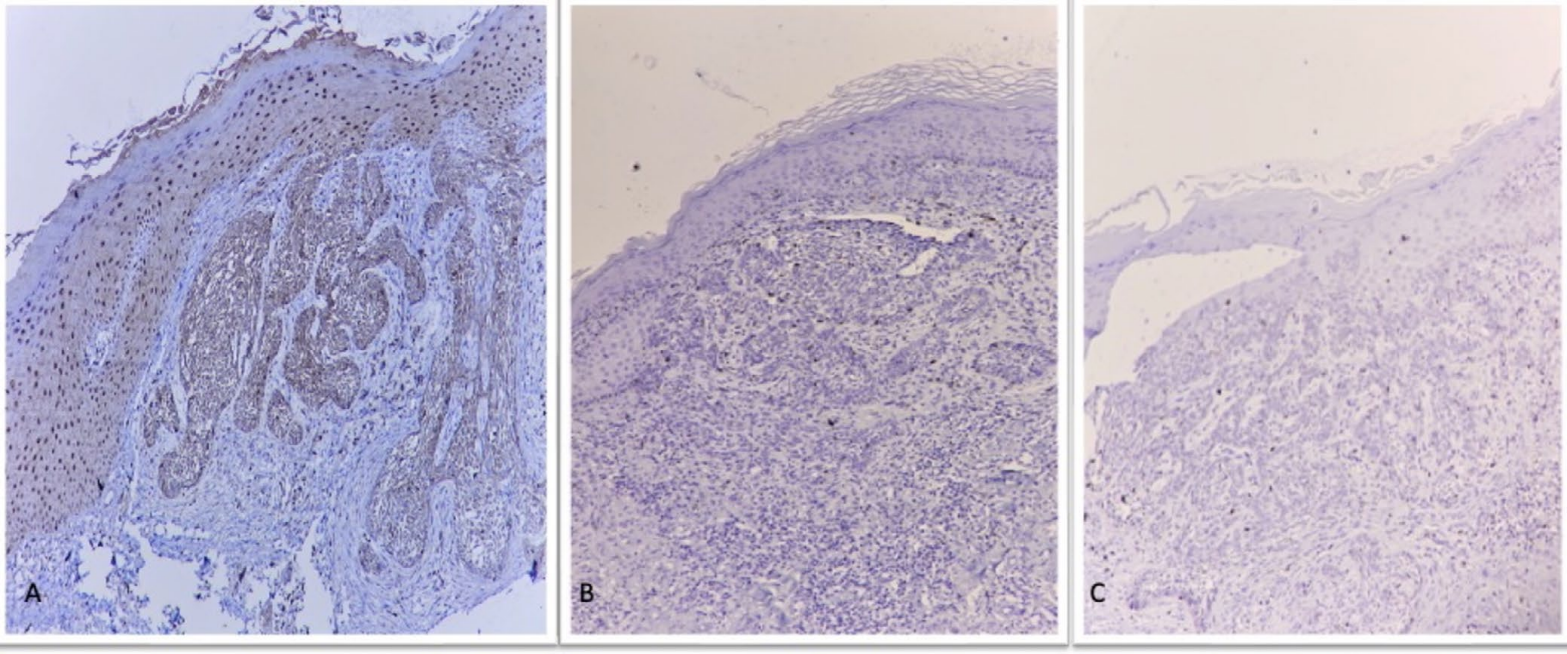
IHC studies: A: Weakly positive BCL‐2. B: Negative SOX10. C: Negative Melan‐A.

Immunohistochemical studies were negative for SOX10, Melan‐A, and CD10, but weakly positive for BCL2, confirming the diagnosis of pigmented basal cell carcinoma(Figure 3).

Pelvic magnetic resonance showed no involvement of the anal sphincter. The patient was referred for complete surgical excision of the lesion.

## 4 Discussion

Among anorectal cutaneous malignancies, perianal BCC is one of the rarest entities.

Approximately 80% of reported patients are men aged 65–75 years [3]. The most common presenting symptoms include anal bleeding, pain, and pruritus.

The pigmented variant of BCC is uncommon, accounting for about 6% of all cases [7]. This case is notable for its unusual histologic subtype, location, and occurrence in a female patient.

The pathogenesis of BCC in non‐sun‐exposed areas remains uncertain. Perianal hair follicles may provide a suitable microenvironment for tumor growth, but anal canal invasion occurs only in the invasive [12].

Other risk factors, including trauma, chronic fistula, and previous radiation exposure, have been described. A 2023 review found associated conditions such as hemorrhoids (2 patients), anal fistula [2], prior radiation [2], prior anal fissure [1], and trauma [1, 21]. These results show that UV exposure is not the sole etiologic factor [21].

In our case, the lesion was located laterally, and the patient had no prior anal fissure. Most reported tumors arise posteriorly, corresponding to the typical site of fissures; however, no definitive correlation has been established [21]. The relatively poor blood supply in the posterior anal region may explain its higher susceptibility.

The differential diagnosis includes squamous cell carcinoma (SCC), melanoma, and basaloid squamous cell carcinoma.

Clinical and histopathologic criteria aid in differentiation. Squamous cell carcinoma often invades the anal canal, whereas BCC rarely does; however, positive BCL‐2 immunoreactivity supported the diagnosis of basal cell carcinoma [4]. In this case, the lesion's pigmentation initially suggested melanoma, but this was excluded by histology and negative Melan‐A and SOX10 staining.

Wide local excision is the treatment of choice for perianal BCC. Achieving tumor‐free margins of 3–5 mm results in cure rates exceeding 95%.

Following the surgery, the National Cancer Institute also recommended a whole‐body examination every six to twelve months after the tumor diagnosis.

This case emphasizes the importance of prompt evaluation of any perianal or genital lesion.

To improve timely care where modesty or gender norms may delay presentation, clinicians should provide culturally sensitive options such as same‐gender examiners and respectful communication. The take‐home message is that patients should not feel ashamed to seek medical evaluation; early examination and diagnosis are essential for the best outcomes.

## Author Contributions

**Farnaz Araghi:** conceptualization, data curation, project administration, resources, writing – original draft. **Sareh Salarinejad:** data curation, formal analysis, investigation. **Azadeh Rakhshan:** investigation, supervision, writing – review and editing. **Hamideh Moravvej Farshi:** conceptualization, supervision, supervision.

## Funding

The authors have nothing to report.

## Ethics Statement

The ethical issues were completely considered to prepare this case report according to our institution's ethical board guidelines. Moreover, this article was prepared regarding the declaration of Helsinki.

## Consent

The patients in this manuscript have given written informed consent to publication of their case details.

## Conflicts of Interest

The authors declare no conflicts of interest.

## Data Availability

Data openly available in a public repository that does not issue DOIs.
